# EP35 Cryoglobulinaemic vasculitis: an uncommon complication of Sjögren’s syndrome

**DOI:** 10.1093/rap/rkaa052.034

**Published:** 2020-11-03

**Authors:** Michael Chen-Xu, Mark Sykes

**Affiliations:** West Suffolk NHS Foundation Trust, Bury St Edmunds, United Kingdom

## Abstract

**Case report - Introduction:**

We present the case of a 70-year-old female with a background of anti-Ro positive Sjögren’s syndrome with difficult sicca symptoms who presented with worsening Raynaud’s, bilateral lower limb petechiae/purpura, fatigue, and progressive peripheral neuropathy.

Blood tests revealed positive cryoglobulins with hypocomplementemia, and nerve conduction studies objectively confirmed the peripheral neuropathy.

The patient was diagnosed with cryoglobulinaemic vasculitis and treated with pulsed intravenous cyclophosphamide and oral prednisolone, which resolved her rash and halted the progression of her symptoms.

Cryoglobulinaemic vasculitis is a rare complication of Sjögren’s syndrome occurring in only 3–4% of patients with the disease.

**Case report - Case description:**

A 70-year-old female with known Sjögren’s syndrome presented with a two-month history of an intermittent red, pin-prick rash affecting her lower legs, worsening fatigue and Raynaud’s, and a progression of longstanding symptoms of pins and needles from her ankles up to her knees, shortly after an acute Epstein-Barr virus infection.

Her Sjögren’s syndrome was diagnosed after she presented with difficult sicca symptoms, fatigue and Raynaud’s phenomenon, and strongly positive ANA and anti-Ro antibodies (>240 u/mL).

Past medical history was notable for microscopic colitis.

On examination, she had bilateral pitting oedema with a purpuric rash affecting both legs. She had reduced sensation to both knees, but with normal power and downgoing plantars. Her joints examined normally. There were no ischaemic changes in her peripheries.

Blood tests showed a positive cryoglobulin consisting of a monoclonal IgM paraprotein with polyclonal lambda light chains. She had a normal kappa: lambda ratio and Bence-Jones proteins. Inflammatory markers were raised (CRP 34mg/L, ESR 93mm/hour), with hypocomplementemia (C4 0.05g/L, normal C3). Otherwise, her full blood count, electrolytes, renal and liver function tests, chest X-ray, urine dipstick, hepatitis serology, ANCA profile, B12, folate and ferritin were unremarkable.

Nerve conduction studies showed a length-dependent, moderately severe sensory motor axonal peripheral neuropathy, which Neurology agreed was due to a vasculitic process.

The patient was diagnosed with a cryoglobulinaemic vasculitis with peripheral nerve involvement secondary to her Sjögren’s syndrome.

This was initially treated with prednisolone 40mg daily, intravenous pulsed cyclophosphamide, which resolved her rash and halted the progression of her peripheral neuropathy. Pregabalin was prescribed for pain relief.

After completing six cycles of cyclophosphamide, the patient was commenced on azathioprine. This was then replaced with mycophenolate due to leukopenia. She was gradually weaned off steroids, and her vasculitis to date remains biochemically and clinically stable.

**Case report - Discussion:**

The presence of the combination of a petechial/purpuric rash on her lower limbs, worsening fatigue and Raynaud’s, and symptoms consistent with a progressive peripheral neuropathy raised the suspicion of a vasculitic process in this patient, which warranted urgent investigation.

She had a type II mixed cryoglobulinemia which is the most common type of cryoglobulinemia found in Sjögren’s syndrome, evidenced by the presence of a monoclonal IgM paraprotein with polyclonal lambda light chains.

Cryoglobulinaemic vasculitis is a systemic vasculitis characterised by the deposition of immune complexes into small vessels, commonly affecting the peripheral nerves, skin, and joints. Clinically, this can manifest with arthralgias/arthritis; constitutional symptoms, such as fatigue and fever; neurologically, with peripheral neuropathies, cranial nerve and central nervous system involvement; and with vascular symptoms, such as petechiae/purpura, skin ulcers, hyperviscosity syndrome, and Raynaud’s. Laboratory features consistent with a diagnosis of cryoglobulinaemic vasculitis aside from the sine qua non of positive cryoglobulins include hypocomplementemia (especially complement C4), positive rheumatoid factor, and a positive serum monoclonal component.

We suspect that her cryoglobulinaemic vasculitis was most likely due to Sjögren’s syndrome, although it could have been triggered by the preceding Epstein-Barr virus infection, as this can be associated with cryoglobulinemia also.

The decision to treat aggressively with pulsed intravenous cyclophosphamide and prednisolone was made given the severity of the patient’s symptoms, especially her progressive peripheral neuropathy. Given the paucity of data in the literature on the management of cryoglobulinaemic vasculitis secondary to rheumatological conditions, cyclophosphamide and prednisolone were chosen as these are proven in the other small vessel vasculitides, such as ANCA-associated vasculitis. This case is of interest as cryoglobulins are found in approximately 7–16% of patients with Sjögren’s syndrome, with cryoglobulinaemic vasculitis seen in only 3–4% of patients with the disease.

**Case report - Key learning points:**

Cryoglobulins are uncommon in Sjögren’s syndrome, occurring in 7–16% of those with the disease. Symptomatic cryoglobulinaemic vasculitis among those with Sjögren’s syndrome is rare, seen in only 3–4% of cases.

The presence of cryoglobulins in Sjögren’s syndrome is of clinical significance, as it is associated with higher global systemic disease activity and extra glandular involvement. Compared to non-cryoglobulinaemic patients with Sjögren’s syndrome, those with cryoglobulinemia are more likely to have lymphadenopathy, constitutional symptoms, peripheral nervous system and pulmonary involvement, and glandular, articular, and cutaneous features of the disease.

The type of cryoglobulinemia found in Sjögren’s syndrome is the mixed type, which are either formed from a monoclonal immunoglobulin (usually IgM) and a polyclonal immunoglobulin (type II), or two polyclonal immunoglobulins (type III). Other conditions associated with mixed cryoglobulinemia include rheumatoid arthritis and systemic lupus erythematosus (SLE), although the most common cause of these is chronic hepatitis C (80–90% of cases). Other causes of mixed cryoglobulinemia include other viral infections, including Epstein-Barr virus and hepatitis B, and certain bacterial and parasitic infections.

Most of the literature on the management of cryoglobulinaemic vasculitis is in the context of patients with this due to chronic hepatitis C and revolves around treating this with the appropriate antiviral therapy. Consequently, the current treatment options for moderate-to-severe cryoglobulinaemic vasculitis secondary to rheumatological conditions are the same as those for the other small vessel vasculitides, using a combination of cyclophosphamide and glucocorticoids to induce remission, and azathioprine as maintenance therapy. In severe cases, plasma exchange and rituximab can also be considered as agents to induce remission in cryoglobulinaemic vasculitis.

